# Structural Features of Eu^3+^ and Tb^3+^-Bipyridinedicarboxamide Complexes

**DOI:** 10.3390/polym14245540

**Published:** 2022-12-18

**Authors:** Anna S. Miroshnichenko, Konstantin V. Deriabin, Artem A. Rashevskii, Vitalii V. Suslonov, Alexander S. Novikov, Ivan S. Mukhin, Regina M. Islamova

**Affiliations:** 1 Institute of Chemistry, St. Petersburg University, 7/9 Universitetskaya Emb., St. Petersburg 199034, Russia; 2 ChemBio Cluster, ITMO University, 49 Kronverksky Pr., St. Petersburg 197101, Russia; 3 Research Institute of Chemistry, Peoples’ Friendship University of Russia (RUDN University), 6 Miklukho-Maklaya Str., Moscow 117198, Russia; 4 Laboratory of Renewable Energy Sources, St. Petersburg Academic University, 8/3 Khlopina Str., St. Petersburg 194021, Russia; 5 High School of Engineering Physics, The Great St. Petersburg Polytechnical University, 29 Polytechnicheskaya Str., St. Petersburg 195251, Russia

**Keywords:** lanthanide complexes, terbium, europium, structural features, photoluminescence, polyethylene glycol

## Abstract

Photoluminescent lanthanide complexes of Eu^3+^ and Tb^3+^ as central atoms and *N^6^*,*N^6^’*-diisopropyl-[2,2′-bipyridine]-6,6′-dicarboxamide as ligand were synthesized. The structure of these complexes was established by single-crystal X-ray diffraction, mass spectrometry, ^1^H and ^13^C nuclear magnetic resonance, ultraviolet-visible, infrared spectroscopy, and thermogravimetry. Bipyridinic ligands provide formation of coordinatively saturated complexes of lanthanide ions and strong photoluminescence (PL). The Eu^3+^- and Tb^3+^-complexes exhibit PL emission in the red and green regions observed at a 340 nm excitation. The quantum yield for the complexes was revealed to be 36.5 and 12.6% for Tb^3+^- and Eu^3+^-complexes, respectively. These lanthanide compounds could be employed as photoluminescent solid-state compounds and as emitting fillers in polymer (for example, polyethylene glycol) photoluminescent materials.

## 1. Introduction

Luminescent lanthanide complexes are receiving a strong interest in their application in optoelectronics [1], photonics [2], amplifiers [3], cell dyes fabrication [4], photocatalysis [5], UV light-sensing or dosimeter materials [6], theranostics [7], and development of fluorescent probes for bioimaging [8]. The mentioned fields associated with lanthanide complexes are due to their narrow emission lines, large Stokes displacement, high quantum yield (QY), and long-lived luminescence lifetimes, as opposed to lanthanide ions directly, which possess the poor light absorption because of Laporte forbidden *f-f* transitions [9].

Among a great range of photoluminescent lanthanide complexes, europium(III) and terbium(III) organic luminophores are broadly investigated, since Eu^3+^ and Tb^3+^ cations have intense pure red and green emission in the visible region [10]. These emission colors are the components of the RGB system [11] that could be used as a light converter for light-emitting diodes and colored displays fabrication.

As mentioned in the ref. [12], 2,2′-bipyridine-6,6′-dicarboxylate ligand in complexes is a highly efficient sensitizer for the “antenna effect”. Thus, the use of 2,2′-bipyridine-6,6′-dicarboxylate ligands, especially amides—*N^6^,N^6^’*-diisopropyl-[2,2′-bipyridine]-6,6′-dicarboxamide (BDCA), in europium and terbium complexes, can lead to a relatively higher QY compared to several europium and terbium complexes with some ligands, including phenanthroline derivatives, thenoyltrifluoroacetone, β-diketonates, substituted pyridine, and 2,2′-bipyridine derivatives [13,14,15,16,17,18,19].

To the best of our knowledge, the photoluminescent properties of lanthanide(III) coordinatively saturated complexes with BDCA have never been studied. The only known similar lanthanide(III)-incorporating polymer-metal complexes (PMCs) are based on polydimethylsiloxanes functionalized by tetradentate bipyridine, functioning as ligands toward Eu^3+^ and Tb^3+^ [11]. Previously, we designed PMCs of Eu^3+^ and Tb^3+^-bipyridinedicarboxamide-*co*-polydimethylsiloxanes, which exhibit a high photoresponse and are applied as flexible, self-healing, and color-tunable photoluminophores in flexible device applications [11]. The main advantage of these PMCs is that they can be mechanically stacked one above another to achieve the desired emission color in the spectral range from green to yellow and red. The external quantum efficiency of these PMCs was good enough, however, it did not exceed 10.5% and 18.3% at a 340 nm excitation in the case of Eu and Tb-PMCs, respectively. These QY values are related to luminescence quenching due to the use of a polymer ligand [20]. Therefore, the use of low-molecular-weight BDCA ligand instead of polysiloxane bipyridine-containing ligand can increase QY compared to ref. [11]. Hence, the development of methods for the synthesis of luminescent complexes based on Eu^3+^, Tb^3+^, and BDCA, with high QY, is a challenging task.

Thus, the aims of the study are to (*i*) synthesize lanthanide complexes with a novel framework by complexation of Eu^3+^ and Tb^3+^ ions with *N^6^,N^6^’*-diisopropyl-[2,2′-bipyridine]-6,6′-dicarboxamide (BDCA) organic ligand, (*ii*) establish their structure by single-crystal X-ray diffraction (XRD), high-resolution electrospray ionization mass spectrometry (HRESIMS), ^1^H, ^13^C NMR, UV-vis, and FTIR spectroscopy, (*iii*) study their photoluminescent properties, (*iv*) incorporation of Eu, Tb-complexes as fillers in polymer films (polyethylene glycol, PEG) for creating luminescent composites, and (*v*) study their thermal stability. All our experimental data and the corresponding discussion are detailed in the following sections.

## 2. Results

### 2.1. Synthesis of Lanthanide Complexes

**Scheme 1:**
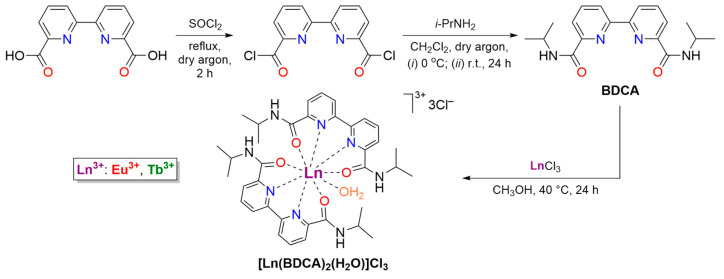
Synthetic scheme of [Eu(BDCA)_2_(H_2_O)]Cl_3_ and [Tb(BDCA)_2_(H_2_O)]Cl_3_.

Lanthanide(III) complexes [Eu(BDCA)_2_(H_2_O)]Cl_3_ and [Tb(BDCA)_2_(H_2_O)]Cl_3_ were synthesized by a three-stage procedure including the two-step synthesis of ligand and its complexation with lanthanide(III) chlorides (Scheme 1).

The BDCA ligand was obtained by the reaction between pre-prepared [2,2′-bipyridine]-6,6′-dicarbonyl dichloride (Scheme 1) and isopropylamine in CH_2_Cl_2_ at room temperature (r.t., 21 °C). The structure of BDCA ligand was established by ^1^H NMR, indicating a ^1^H NMR signal of amide groups (N***H***C(=O) at δ = 8.62 ppm, ^13^C NMR (spectra are illustrated in Figure S1 in the Supplementary Material), HRESIMS, and single-crystal XRD (Figures S2 and S3, Table S1).

In the last stage, the complexation between BDCA and dry lanthanide(III) chlorides (EuCl_3_ and TbCl_3_) with metal–ligand molar ratio of 1:2 was conducted in a CH_3_OH solution at 40 °C for 24 h in order to obtain complexes [Eu(BDCA)_2_(H_2_O)]Cl_3_ and [Tb(BDCA)_2_(H_2_O)]Cl_3_. The synthesized complexes are soluble in DMSO and alcohols, especially EtOH.

### 2.2. Structure of Lanthanide Complexes

**Figure 1:**
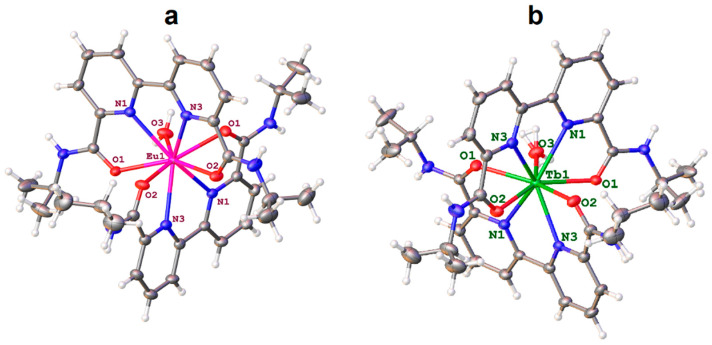
Molecular structures of the inner spheres of [Eu(BDCA)_2_(H_2_O)]Cl_3_ (**a**) and [Tb(BDCA)_2_(H_2_O)]Cl_3_ (**b**) with thermal ellipsoids shown at the 50% probability level. The Cl^–^ anions and water molecules of the outer spheres of the complexes are omitted for better representability.

**Figure 2:**
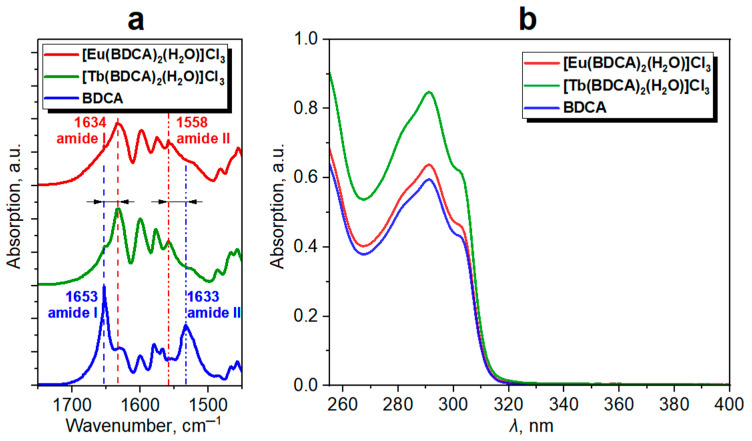
Spectra of [Eu(BDCA)_2_(H_2_O)]Cl_3_, [Tb(BDCA)_2_(H_2_O)]Cl_3_, and BDCA: FTIR in KBr (**a**) and UV-vis in DMSO (**b**).

The synthesized complexes were then characterized by XRD, HRESIMS, FTIR, and UV-vis (Figure 1 and Figure 2 and Figures S4 and S5, Table S1). The single-crystal XRD data indicated that lanthanide(III) coordination with BDCA induces bonding between Ln–N_Bipy_ and Ln−O (Ln = Eu, Tb, Figure 1, for details, see Experimental section). The complexation between BDCA and Ln^3+^ should be conducted via *O*,*N*,*N*,*O*-chelating moieties ligation. Thus, considering the coordination number 9 of Ln^3+^, the only possible conclusion in the metal–ligand molar ratio is 1:2. Europium and terbium complexes have the same geometry of the nine-vertex polyhedron with distorted pentagonal coordination formed by oxygen atoms in the equatorial position and nitrogen atoms along the tetragonal tetrahedron in apical positions. This coordination polyhedron contains crystallographic 2-fold axes passing through the metal atom and the coordinated water molecule.

In the HRESIMS spectra, the [Eu(BDCA)_2_]^3+^ (*m/3* = 268.4233) and [Tb(BDCA)_2_]^3+^ ions (*m/3* = 270.4241) were indicated with their characteristic isotopic distribution (Figures S4 and S5), respectively. In the FTIR spectra of [Eu(BDCA)_2_(H_2_O)]Cl_3_ and [Tb(BDCA)_2_(H_2_O)]Cl_3_, the amide I *ν*(C=O) band was shifted from 1653 to 1631 cm^−1^ in comparison with the BDCA (Figure 2a). According to the refs. [11,21,22,23], this band shift can be attributed to the involvement of the C=O group in the coordination.

As a result, [Eu(BDCA)_2_(H_2_O)]Cl_3_ and [Tb(BDCA)_2_(H_2_O)]Cl_3_ complexes were synthesized by a complexation of EuCl_3_ and TbCl_3_ with BDCA ligand. Considering all the spectral, XRD, and HRESIMS data, we conclude that the ligation of BDCA occurred via Ln–N_Bipy_ and Ln−O bonding. The complexes are heteroleptic, composed of Ln^3+^, two BDCA ligands, and one molecule of coordinated H_2_O.

### 2.3. Theoretical Calculations of HOMO–LUMO Energy Gaps

The energies for the coordination bonds Ln–O and Ln–N_Bipy_ (Ln = Eu, Tb) were computed by appropriate quantum chemical calculations at the ωB97XD/DZP-DKH level of theory, followed by the topological analysis of the electron density distribution (for details, see the Computational Details section in the Supplementary Material). Our results are summarized in Table S2, while the model structures, the contour line diagrams of the Laplacian of electron density distribution *∇^2^ρ(r)*, bond paths, selected zero-flux surfaces, visualization of electron localization function (ELF), reduced density gradient analyses referring to coordination bonds Ln–O and Ln–N_Bipy_, and Cartesian atomic coordinates of [Eu(BDCA)_2_(H_2_O)]Cl_3_ and [Tb(BDCA)_2_(H_2_O)]Cl_3_ model structures are shown in Figures S6−S8 and Table S3 (Supplementary Material). Thus, Ln–N coordination bonds exhibited a slightly longer bond length (c.a. 2.5 Å) and energy values (c.a. 11–12 kcal·mol^−1^) lower than the Ln–O bonds (*l* = 2.4 Å, *E_int_* = 15–16 kcal·mol^−1^). Two BDCA ligands are similarly ligated to the Ln^3+^ center in terms of binding energies.

The calculated HOMO–LUMO energy gaps (*E_H-L_*) in model structures [Eu(BDCA)_2_(H_2_O)]Cl_3_ and [Tb(BDCA)_2_(H_2_O)]Cl_3_ are 7.50 and 8.17 eV, respectively (theoretical calculations are presented in Section S4.2 in the Supplementary Material).

### 2.4. Band Gap Estimation by UV-Vis Absorption Study

In the UV-vis spectra of [Eu(BDCA)_2_(H_2_O)]Cl_3_ and [Tb(BDCA)_2_(H_2_O)]Cl_3_ (Figure 2b), a peak at 291 nm (with two shoulders at 283 and 302 nm) is observed, which corresponds to ligand-to-metal charge transfer (LMCT) (theoretical modeling of UV-vis absorption spectra is presented in Section S4.3 in the Supplementary Material, Figure S9). The UV-vis spectra of the complexes were similar to the spectral shape of the free ligand, since the π–π* state energy was probably not influenced by the coordination sphere of the metal [2,15,24,25,26].

**Figure 3:**
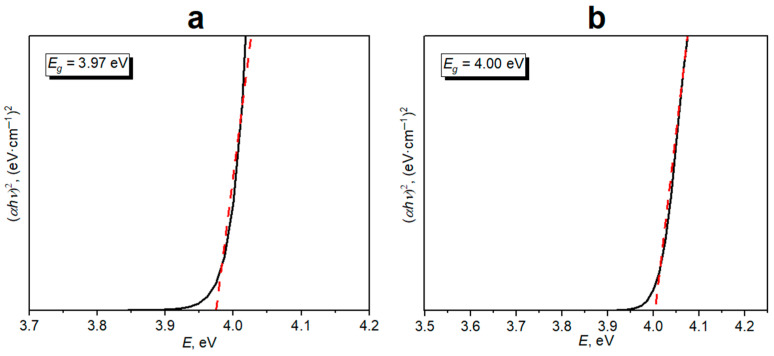
Tauc’s plots for band gap determination of [Eu(BDCA)_2_(H_2_O)]Cl_3_ (**a**) and [Tb(BDCA)_2_(H_2_O)]Cl_3_ (**b**).

Optical band gap of [Eu(BDCA)_2_(H_2_O)]Cl_3_ and [Tb(BDCA)_2_(H_2_O)]Cl_3_ was estimated from absorption spectral data using Tauc’s relation (Equation 1), which is represented below:

***α**E* = *A*(*E* − *E_g_*)*^m^*, (1)

where ***α***, *E,* and *m* represent absorption coefficient, photon’s energy, and optical parameters, respectively [27]. Tauc’s profiles were plotted as (*αhυ*) ^2^–energy (*E*). Extrapolation of the tangent down to the *x*-axis provides the value of the band gap. Figure 3 shows the Tauc’s profiles of complexes, which demonstrate a similar band gap *E_g_* = 4.0 eV of [Eu(BDCA)_2_(H_2_O)]Cl_3_ and [Tb(BDCA)_2_(H_2_O)]Cl_3_.

As a result, optical band gap values gained from absorption spectral data using Tauc’s plot (*E_g_* = 4.0 eV) is c.a. two times lower than calculated *E_H-L_* (7.50 and 8.17 eV).

The calculated by the density-functional theory method (DFT) HOMO–LUMO gap gives only an approximation of the band gap and does not take into account the formation of electron-hole semi-particle in the excited state [28]. Thus, the optical gap obtained by Tauc’s relation from the UV-vis spectrum corresponds to the energy of the lowest electronic transition and could be substantially lower than *E_H-L_.*

### 2.5. Luminescent Properties

The use of 2,2′-bipyridine-6,6′-dicarboxylate sensitizer provides efficient energy transfer from an excited state of ligand to Ln^3+^ excited state, which leads to energy transitions ^5^D_4_→^7^F_J_ (*J* = 6–3) for Tb^3+^ and ^5^D_0_→^7^F_J_ (*J* = 0–4) for Eu^3+^ [11,12]. As noticed in the ref. [29], 2,2′-bipyridine-6,6′-dicarboxylate is a highly efficient sensitizer for the “antenna effect”.

**Figure 4:**
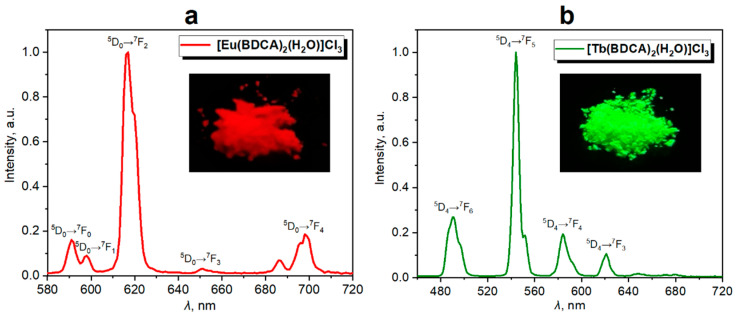
PL emission spectra of [Eu(BDCA)_2_(H_2_O)]Cl_3_ (**a**) and [Tb(BDCA)_2_(H_2_O)]Cl_3_ (**b**).

Emission spectra of [Eu(BDCA)_2_(H_2_O)]Cl_3_ and [Tb(BDCA)_2_(H_2_O)]Cl_3_ under UV light excitation (at excitation wavelength *λ_ex_* = 340 nm, excitation spectra are illustrated in Figure S10) [11] were acquired (Figure 4). Typical energy transitions of Tb^3+^ and Eu^3+^ were observed in PL emission spectra as signals of their characteristic wavelengths.

The studied complexes demonstrate red and green phosphorescence according to acquired PL lifetimes 2.9 and 4.9 ms for [Eu(BDCA)_2_(H_2_O)]Cl_3_ and [Tb(BDCA)_2_(H_2_O)]Cl_3_, respectively (Figure S11).

QY upon direct excitation of the Ln^3+^ ion is determined mainly by the probability of nonradiative processes. For free ions, the probability of nonradiative transitions is smaller, and the energy gap is larger between the resonant level of the Ln^3+^ ion and the first level of the main multiplet. QY values for [Eu(BDCA)_2_(H_2_O)]Cl_3_ and [Tb(BDCA)_2_(H_2_O)]Cl_3_ complexes are presented in Table 1. According to the ref. [12], the difference in PL between complexes can be explained by Δ*E* = 12,300 cm^−1^ (^5^D_0_→^7^F_0_ ) and 14,800 cm^−1^ (^5^D_4_→^7^F_6_) for Eu^3+^ and Tb^3+^.

The QY values for [Eu(BDCA)_2_(H_2_O)]Cl_3_ and [Tb(BDCA)_2_(H_2_O)]Cl_3_ are expectedly higher than that of corresponding reported Eu^3+^ and Tb^3+^-incorporating PMCs [11] (Table 1) and some other bipyridine derivatives of Eu^3+^ and Tb^3+^ [2,14,15]. The increase in QY in complexes is associated with use of low-molecular-weight BDCA ligand in studied complexes instead of bipyridine-incorporating polymer ligand [11]. PMCs contain polymer ligands with the number-average molecular weight of 45,000–50,000 [21] compared to BDCA ligand with a molecular weight of 326, which leads to luminescence quenching [20,30].

**Figure 5:**
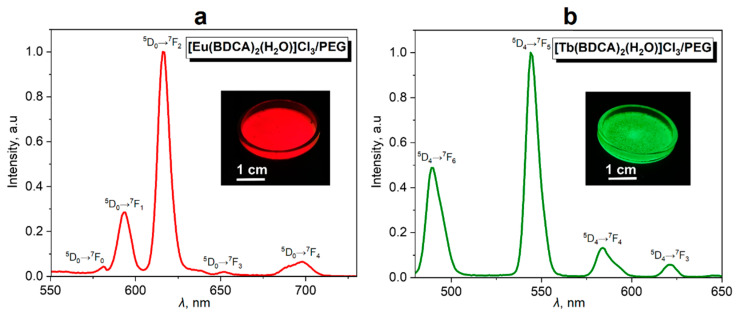
PL emission spectra of [Eu(BDCA)_2_(H_2_O)]Cl_3_ (**a**) and [Tb(BDCA)_2_(H_2_O)]Cl_3_ (**b**) encapsulated in PEG at a concentration of 10^−3^ mg·mL^−1^ under UV-light (340 nm).

The [Eu(BDCA)_2_(H_2_O)]Cl_3_ and [Tb(BDCA)_2_(H_2_O)]Cl_3_ can retain a high QY even after encapsulation in a semitransparent polymer matrix (Figure 5). Taking into account the good solubility of the complexes (Section 2.1. Synthesis of lanthanide complexes), soluble in EtOH PEG was chosen as the polymer matrix. The QY values of [Eu(BDCA)_2_(H_2_O)]Cl_3_ and [Tb(BDCA)_2_(H_2_O)]Cl_3_ encapsulated in PEG were 11.2 and 25.3%, respectively. This was compared to the pure [Eu(BDCA)_2_(H_2_O)]Cl_3_ in the emission spectra signal at 580 nm, which corresponds to the forbidden transition ^5^D_0_→^7^F_0_, which disappears after encapsulation in PEG. The intensity increases for the signals ^5^D_0_→^7^F_0_ (592 nm) and ^5^D_4_→^7^F_6_ (485 nm), which correspond to energy transitions from excited state to ground state of Eu^3+^ and Tb^3+^, respectively. More information about excitation spectra and PL lifetimes are described in the Supplementary Material (Section S5—“Luminescent properties”, Figures S10 and S11).

### 2.6. Thermal Stability

**Figure 6:**
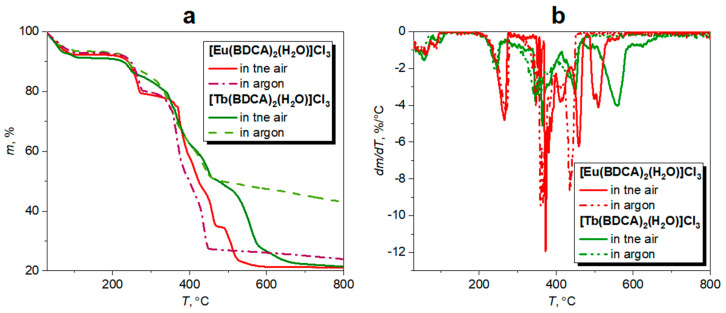
TG (**a**) and differential TG curves (**b**) of [Eu(BDCA)_2_(H_2_O)]Cl_3_ and [Tb(BDCA)_2_(H_2_O)]Cl_3_.

TG and differential TG curves of [Eu(BDCA)_2_(H_2_O)]Cl_3_ and [Tb(BDCA)_2_(H_2_O)]Cl_3_ indicate some steps of mass loss (Figure 6): 5–7% mass loss at 100 °C (dehydration), 15–20% at 280 °C, and 40% at 480 °C. The final thermal destruction of coordination centers of [Eu(BDCA)_2_(H_2_O)]Cl_3_ and [Tb(BDCA)_2_(H_2_O)]Cl_3_ probably starts at 380 °C.

## 3. Materials and Methods

### 3.1. Materials

[2,2′-bipyridine]-6,6′-dicarboxylic acid (97%, BLD Pharm, Shanghai, China), Et_3_N (99%, Abcr GmbH, Karlsruhe, Germany), and isopropylamine (99%, Abcr GmbH, Karlsruhe, Germany) were purchased from commercial suppliers, and their purity was checked by ^1^H NMR. Ultradry EuCl_3_ (99.99%) and TbCl_3_ (99.99%) were acquired from ChemCraft Ltd. (Kaliningrad, Russia). SOCl_2_ and CH_2_Cl_2_ (99%, Vecton, St. Petersburg, Russia) were distilled under argon (though the latter was also over P₂O₅). CH_3_OH (99%, Vecton, St. Petersburg, Russia) was dried and distilled over (OCH_3_)_2_Mg prior to use. Et_2_O (99%, Vecton, St. Petersburg, Russia) was freshly distilled over sodium/benzophenone under an argon atmosphere prior to use. DMSO (99%, Reachem, Moscow, Russia), EtOH (99%, Himprod, Ekaterinburg, Russia), and PEG polymer (*M_n_* = 10 000; 1.2 g·mL; Merck KGaA, St. Louis, MO, USA) were used as received.

### 3.2. Methods

The NMR spectra have been recorded on a Bruker Avance III 400 spectrometer (Germany) in DMSO-*d_6_* at r.t. (at 400 MHz for ^1^H and 100 MHz for ^13^C). ^13^C NMR spectra were recorded with ^1^H decoupling. The chemical shifts are given in δ-values [ppm] and refer to the residual signals of non-deuterated DMSO: δ 2.50 (^1^H) and 39.5 (^13^C). The following abbreviations were used to designate multiplicities: s = singlet, d = doublet, t = triplet, m = multiplet, br = broad, and dd = doublet of doublets. FTIR spectra were recorded on a Shimadzu IRAffinity-1 FTIR spectrophotometer (Kyoto, Japan) in KBr pellets. The measurements were carried out at RT in the wavenumber range of 400–4000 cm^−1^. The following abbreviations of the absorption bands are used to designate intensity: s—strong, m—medium, and w—weak. UV-vis spectra were recorded on a Shimadzu UV-1800 spectrophotometer (Kyoto, Japan) in DMSO. The measurements have been carried out at RT using a quartz cell 1 cm wide in the wavelength range of 250–800 nm. HRESIMS was conducted on a Bruker Maxis HRMS ESI QTOF spectrometer equipped with an electrospray ionization source. The analyzed samples had been dissolved beforehand in pure CH_3_OH for the HRESIMS measurements. The instrument has been operated at a positive ion mode using the *m/z* range of 50–400. The most intense peak in the isotopic pattern is noted.

Crystallographic data for all crystals were obtained using Rigaku Oxford Diffraction «SuperNova» (Tokyo, Japan) (in the case of BDCA) and Rigaku Oxford Diffraction «Synergy XtaLAB» (Tokyo, Japan) (in the case of [Eu(BDCA)_2_(H_2_O)]Cl_3_ and [Tb(BDCA)_2_(H_2_O)]Cl_3_) diffractometers with monochromated micro-focus CuKα (*λ* = 1.54184) X-ray sources. All crystals were kept at 100 K during all data collection. Crystal structures were solved using ShelXT [31] structure solution program and refined by means using ShelXL [32] structure refinement program incorporated in the Olex2 program package [33]. All crystallographic data for this paper can be obtained free of charge via the Cambridge Crystallographic Database.

PL spectra were recorded on a Horiba Fliorolog-3 spectrofluorometer (Jobin Yvon Technology, Bensheim Germany) at r.t. QY measurements were performed using a Shimadzu RF-6000 spectrofluorometer (Kyoto, Japan) with an integrating sphere (101 mm in diameter). PL lifetime steady state measurements were performed via Horiba Fliorolog-3 spectrofluorometer with the impulse xenon lamp with a power of 150 W as an excitation source.

TG was carried out on a NETZSCH TG 209F1 Libra TGA209F1D0024 analyzer (Selb, Germany) in the air and inert (argon) atmosphere. The samples were heated from 50 to 800 °C at a heating rate of 10 °C∙min^−1^.

### 3.3. Synthetic Procedures

#### 3.3.1. [2,2′-Bipyridine]-6,6′-Dicarbonyl Dichloride

A round-bottom flask, which was purged with argon, and equipped with a reflux condenser, was charged with [2,2′-bipyridine]-6,6′-dicarboxylic acid (1.0 g, 4.1 mmol), 10 µL of Et_3_N, and freshly distilled SOCl_2_ (250 mL). The mixture had been refluxed for 2 h under a constant argon flow until complete dissolution of the dicarboxylic acid and then cooled to r.t. The excess SOCl_2_ was removed at r.t. under reduced pressure for 3 h to obtain powdery dicarbonyl dichloride. Yield: 1.14 g (99%); beige crystals; mp 288 °C.

#### 3.3.2. *N^6^,N^6^’*-Diisopropyl-[2,2′-Bipyridine]-6,6′-Dicarboxamide (BDCA)

A freshly prepared 2,2′-bipyridine-6,6′-dicarbonyl dichloride (1.14 g, 4.06 mmol) solution in dry CH_2_Cl_2_ (10 mL) was added to isopropylamine (2.39 g, 40.6 mmol) solution in dry CH_2_Cl_2_ (20 mL) by drops at 0 °C under an argon atmosphere. The reaction mixture was stirred at r.t. for 24 h and then filtered. Afterwards, it was filtered and washed with distilled water (3 × 100 mL). The residue was dried under vacuum at 65 °C and washed with Et_2_O to afford pure BDCA. Yield: 0.93 g (70%); beige powder. ^1^H NMR (DMSO-d_6_, δ): 1.28 (d, *J* = 6.6 Hz, 12H, CH(C***H_3_***)_2_), 4.23 (m, *J_1_* = 6.6 Hz, *J_2_* = 8.5 Hz, 2H, C***H***(CH_3_)_2_), 8.13 (dd, *J_1_* = 7.7 Hz, *J_2_* = 1.3 Hz, 2H, 5,5′-H-Bipy), 8.19 (t, *J_1_* = 7.7 Hz, *J_2_* = 7.7 Hz, 2H, 4,4′-H-Bipy), 8.62 (d, *J* = 8.5 Hz, 2H, N***H***C(=O)), 8.98 (dd, *J_1_* = 7.7 Hz, *J_2_* = 1.3 Hz, 2H, 3,3′-H-Bipy). ^13^C NMR (DMSO-d_6_, δ): 22.7 (CH(***C***H_3_)_2_), 41.4 (***C***H(CH_3_)_2_), 123.0 (3,3′-C_Bipy_), 124.3 (5,5′-C_Bipy_), 139.3 (4,4′-C_Bipy_), 150.4 (6,6′-C_Bipy_), 153.8 (2,2′-C_Bipy_), 163.2 (C=O). FTIR (KBr, selected bands, *ν*, cm^−1^): 3303 (s; *ν*(N–H)), 1653 (s; *ν*_amide I_ (C=O)), 1533 (s; *ν*_amide II_ (N–H)). UV-vis (DMSO, *λ*_max_, nm): 302 (C=O, n→π*), 291 (Bipy, π→π*), and 282 nm (Bipy, π→π*). HRESIMS^+^: calculated for C_18_H_22_N_4_O_2_ 349.1640, found *m/z* 349.1630 [M+Na]^+^. Single crystals of BDCA were grown from CH_3_OH solution over a one-week period for XRD. Crystal lattice parameters: *a* = 9.2043(6), *b* = 11.4313(5), *c* = 9.6843(6); *α* = 90°, *β* = 116.612(8)°, and *γ* = 90°; monoclinic, space group *P2_1_/c* (14), temperature 100 K. CCDC number: 2215221.

#### 3.3.3. [Eu(BDCA)_2_(H_2_O)]Cl_3_

BDCA (465 mg, 1.42 mmol) was placed in a round-bottom flask and dissolved in anhydrous CH_3_OH (20 mL). A solution of dry EuCl_3_ (175 mg, 0.68 mmol) in 2 mL of anhydrous CH_3_OH was slowly added dropwise to the resulting mixture. The solution was stirred at 40 °C for 24 h, and the solvent was removed by rotary evaporation at 60 °C. The obtained residue was then washed with Et_2_O (3 × 50 mL). Yield: 608 mg (95%); beige crystals. FTIR (KBr, selected bands, *ν*, cm^−1^): 3410 (s; *ν*(O–H)), 3220 (s; *ν*(N–H)), 1634 (s; *ν*(C = O--Eu)), 1558 (s; *ν*_amide II_ (N–H)), 1456 (m; *ν*(Py)). UV-vis (DMSO, *λ*_max_, nm): 302 (C=O, n→π*), 291 (LMCT), 283 nm (LMCT). HRESIMS^+^: calculated for C_36_H_44_N_8_EuO_4_^3+^ 268.4233, found *m/z* 268.4232 [M–3Cl]^3+^. Single crystals of [Eu(BDCA)_2_(H_2_O)]Cl_3_ were grown from EtOH solution over a two-week period for XRD. Crystal lattice parameters: *a* = 11.2713(2), *b* = 15.3222(2), and *c* = 14.2917(2); *α* = 90°, *β* = 106.511(2)°, and *γ* = 90°; monoclinic, space group *P2/c* (13), temperature 100 K. Selected bond lengths (Å): Eu1–O1 2.4137(19), Eu1–O2 2.4019(19), Eu1–O3(H_2_O) 2.463(3), Eu1–N1 2.536(2), and Eu1–N3 2.541(2). Selected bond angles (°): O1–Eu1–N1 64.25(7), N1–Eu1–N3 62.37(7), O2–Eu1–N3 64.30(7), and O1–Eu1–O3(H_2_O) 70.26(5). CCDC number: 2215232.

#### 3.3.4. [Tb(BDCA)_2_(H_2_O)]Cl_3_

BDCA (465 mg, 1.42 mmol) was placed in a round-bottom flask and dissolved in anhydrous CH_3_OH (20 mL). A solution of dry TbCl_3_ (180 mg, 0.68 mmol) in 2 mL of anhydrous CH_3_OH was slowly added dropwise to the resulting mixture. The solution was stirred at 40 °C for 24 h, and the solvent was removed by rotary evaporation at 60 °C. The obtained residue was then washed with Et_2_O (3 × 50 mL). Yield: 606 mg (94%); beige crystals. FTIR (KBr, selected bands, *ν*, cm^−1^): 3416 (s; *ν*(O–H)), 3222 (s; *ν*(N–H)), 1634 (s; *ν*(C=O--Tb)), 1558 (s; *ν*_amide II_ (N–H)), 1457 (m; *ν*(Py)). UV-vis (DMSO, *λ*_max_, nm): 302 (C=O, n→π*), 291 (LMCT), 283 nm (LMCT). HRESIMS^+^: calculated for C_36_H_44_N_8_TbO_4_^3+^ 270.4241, found *m/z* 268.4232 [M–3Cl]^3+^. Single crystals of [Eu(BDCA)_2_(H_2_O)]Cl_3_ were grown from EtOH solution over a two-week period for XRD. Crystal lattice parameters: *a* = 11.2666(3), *b* = 15.2986(3), *c* = 14.2345(3); *α* = 90°, *β* = 106.335(3)°, and *γ* = 90°; monoclinic, space group *P2/c* (13), temperature 100 K. Selected bond lengths (Å): Tb1–O1 2.390(2), Tb1–O2 2.3802(19), Tb1–O3(H_2_O) 2.445(3), Tb1–N1 2.512(2), and Tb1–N3 2.517(2). Selected bond angles (°): O1–Tb1–N1 64.76(7), N1–Tb1–N3 62.65(8), O2–Tb1–N3 64.88(7), and O1–Tb1–O3(H_2_O) 70.18(5). CCDC number: 2215233.

### 3.4. Encapsulation of Lanthanide Complexes in a PEG Matrix

A solution of the corresponding lanthanide(III) chloride in EtOH (0.5 mL, concentration 2 mg·mL^−1^) was mixed with a solution of PEG (1 g) in EtOH (2.0 mL). The resulting mixture was intensely stirred at r.t. for 10 min and then poured into a glass Petri dish and dried at 70 °C for 1 h in order to remove EtOH from the polymer product. For PL studies, thin films were peeled off the Petri dish via a razor blade.

## 4. Conclusions

The red and green-emitting luminescent Eu^3+^ ([Eu(BDCA)_2_(H_2_O)]Cl_3_) and Tb^3+^ ([Tb(BDCA)_2_(H_2_O)]Cl_3_) bipyridine complexes were synthesized in three steps using the pre-prepared *N^6^*,*N^6^’*-diisopropyl-[2,2′-bipyridine]-6,6′-dicarboxamide (BDCA) ligand and anhydrous LnCl_3_ (Ln = Eu, Tb) as reactants.

The structure of [Eu(BDCA)_2_(H_2_O)]Cl_3_ and [Tb(BDCA)_2_(H_2_O)]Cl_3_ was established by XRD, HRESIMS, FTIR, and UV-vis spectroscopy. The XRD data indicated that Ln(III) coordination with BDCA induce bonding between Ln−N and Ln−O. Amide I *ν*(C=O) band displacements in FTIR spectra are related to the involvement of the C=O group in the coordination that provides the formation of coordinatively saturated complexes.

HOMO–LUMO energy gaps (*E_g_*) estimated from crystallographic data by QTAIM analysis, are 7.50 and 8.17 eV for [Eu(BDCA)_2_(H_2_O)]Cl_3_ and [Tb(BDCA)_2_(H_2_O)]Cl_3_, respectively. Optical band gap value of lanthanide complexes gained from absorption spectral data using Tauc’s plot was *E_g_* = 4.0 eV.

Structural features of [Eu(BDCA)_2_(H_2_O)]Cl_3_ and [Tb(BDCA)_2_(H_2_O)]Cl_3_ provide strong PL of lanthanides in the red and green spectral range, respectively, with sharp lines of high intensity. The QY values of [Eu(BDCA)_2_(H_2_O)]Cl_3_ and [Tb(BDCA)_2_(H_2_O)]Cl_3_ complexes are 12.6% and 36.5% at *λ_ex_* = 340 nm excitation, respectively, and are higher compared to previously reported PMCs [11].

We demonstrated that the complexes can be utilized as emitting fillers in polymer composites, especially in the PEG matrix. The QY values of [Eu(BDCA)_2_(H_2_O)]Cl_3_ and [Tb(BDCA)_2_(H_2_O)]Cl_3_ encapsulated in PEG are 11.2 and 25.3%, respectively.

Hence, we report on novel bipyridinic lanthanide complexes, which could be further employed as photoluminescent solid-state compounds and as emitting fillers in polymer photoluminescent materials for optoelectronic devices.

## Figures and Tables

**Table 1:** QY values for [Eu(BDCA)_2_(H_2_O)]Cl_3_ and [Tb(BDCA)_2_(H_2_O)]Cl_3_ complexes compared to corresponding reported PMCs [11] at *λ_ex_* = 340 nm.

Complex	QY, %	QY of Corresponding Reported PMCs [11], %	QY of Encapsulated Complexes in PEG, %
[Eu(BDCA)_2_(H_2_O)]Cl_3_	12.6	10.5	11.2
[Tb(BDCA)_2_(H_2_O)]Cl_3_	36.5	18.3	25.3

## Data Availability

Data is contained within the article or supplementary material. The data presented in this study are available in https://www.mdpi.com/article/10.3390/polym14245540/s1. All crystallographic data for this paper can be obtained free of charge via the Cambridge Crystallographic Database (CCDC numbers: 2215221, 2215232, and 2215233).

## Supplementary Materials

The following supporting information can be downloaded at: https://www.mdpi.com/article/10.3390/polym14245540/s1, Figure S1: ^1^H (a) and ^13^C NMR (b) spectrum of BDCA ligand; Figure S2: Molecular structures BDCA with thermal ellipsoids shown at the 50% probability level. The molecules of solvents are omitted for better representability; Table S1: Crystallographic information for structures of BDCA, [Eu(BDCA)_2_(H_2_O)]Cl_3_, and [Tb(BDCA)_2_(H_2_O)]Cl_3_.; Figure S3:HRESIMS spectra of BDCA ligand; Figure S4: HRESIMS spectrum of [Eu(BDCA)_2_(H_2_O)]Cl_3_ complex; Figure S5: HRESIMS spectrum of [Tb(BDCA)_2_(H_2_O)]Cl_3_ complex; Figure S6: Model structures [Eu(BDCA)_2_(H_2_O)]Cl_3_ (a) and [Tb(BDCA)_2_(H_2_O)]Cl_3_ (b). Length of coordination bonds Ln–O and Ln–N_Bipy_ (Ln = Eu, Tb) are indicated in Å; Table S2: Values of the density of all electrons — *ρ(r)*, Laplacian of electron density — *∇^2^ρ(r)* and appropriate λ_2_ eigenvalues, energy density — *H_b_*, potential energy density — *V(r)*, Lagrangian kinetic energy — *G(r)*, and electron localization function — *ELF* (a.u.) at the bond critical points (3, −1), corresponding to coordination bonds Ln–O and Ln–N_Bipy_ (Ln = Eu, Tb) in model structures [Eu(BDCA)_2_(H_2_O)]Cl_3_ and [Tb(BDCA)_2_(H_2_O)]Cl_3_, as well as estimated energies for these contacts *E_int_* (kcal·mol^−1^) and bond lengths — *l* (Å); Figure S7: Contour line diagram of the Laplacian of electron density distribution *∇^2^ρ(r)*, bond paths, and selected zero-flux surfaces (left panel), visualization of electron localization function (ELF, center panel) and reduced density gradient (RDG, right panel) analyses referring to coordination bonds Eu–O and Eu–N in model structure [Eu(BDCA)_2_(H_2_O)]Cl_3_. Bond critical points (3, −1) are shown in blue, nuclear critical points (3, −3) — in pale brown, ring critical points (3, +1) — in orange, bond paths are shown as pale brown lines, length units — Å, and the color scale for the ELF and RDG maps is presented in a.u.; Figure S8: Contour line diagram of the Laplacian of electron density distribution *∇^2^ρ(r)*, bond paths, and selected zero-flux surfaces (left panel), visualization of electron localization function (ELF, center panel) and reduced density gradient (RDG, right panel) analyses referring to coordination bonds Tb–O and Tb–N in model structure [Tb(BDCA)_2_(H_2_O)]Cl_3_. Bond critical points (3, −1) are shown in blue, nuclear critical points (3, −3) — in pale brown, ring critical points (3, +1) — in orange, bond paths are shown as pale brown lines, length units — Å, and the color scale for the ELF and RDG maps is presented in a.u.; Figure S9: TD-DFT simulated UV-Vis spectrum of model structure [Eu(BDCA)_2_(H_2_O)]Cl_3_; Table S3: Cartesian atomic coordinates for model structures [Eu(BDCA)_2_(H_2_O)]Cl_3_ and [Tb(BDCA)_2_(H_2_O)]Cl_3_; Figure S10: Excitation spectra of raw [Eu(BDCA)_2_(H_2_O)]Cl_3_ (a), [Tb(BDCA)_2_(H_2_O)]Cl_3_ (b), and the encapsulated complexes in PEG (c, d); Figure S11: Luminescence lifetime decay for [Eu(BDCA)_2_(H_2_O)]Cl_3_ (a), Tb(BDCA)_2_(H_2_O)]Cl_3_ (b), and the encapsulated complexes in PEG (c, d).
