# Supplementary material for: Structural Features of Eu3+ and Tb3+-Bipyridinedicarboxamide Complexes
Source: Polymers (Basel). 2022 Dec 18;14(24):5540. doi: 10.3390/polym14245540 (PMC9786332; doi:10.3390/polym14245540)
Supplement: Supplementary file 1 [file polymers-14-05540-s001.zip › polymers-2089695-supplementary.pdf]

Supplementary Material

Structural features of Eu^{3+} and Tb^{3+}-bipyridinedicarboxamide complexes

Anna S. Miroshnichenko,[1,2†] Konstantin V. Deriabin,[1†] Artem A. Rashevskii,[1] Vitalii V. Suslonov,[1] Alexander S. Novikov,[1,3] Ivan S. Mukhin[1,2,4], and Regina M. Islamova[1*]

[1] Saint Petersburg State University, 7/9 Universitetskaya Emb., St. Petersburg, 199034, Russia;
[2] ITMO University, 49 Kronverksky Pr., St. Petersburg, 197101, Russia;
[3] Peoples' Friendship University of Russia (RUDN University), 6 Miklukho-Maklaya Str., Moscow, 117198, Russia;
[4] St. Petersburg Academic University, 8/3 Khlopina Str., St. Petersburg, 194021, Russia.
† These authors contributed equally.
* Correspondence: r.islamova@spbu.ru (R.M.I.).

Content

1. NMR spectra S1
2. X-ray diffraction S2
3. Mass-spectra S4
4. Theoretical calculations S6
   4.1. Theoretical calculations of the coordination bonding energies S6
   4.2. Theoretical calculations of HOMO–LUMO energy gaps S10
   4.3. Theoretical modeling of UV-vis absorption spectra S10
   4.4. Computational details S10
5. Luminescent properties S17
6. References S19

1. NMR spectra

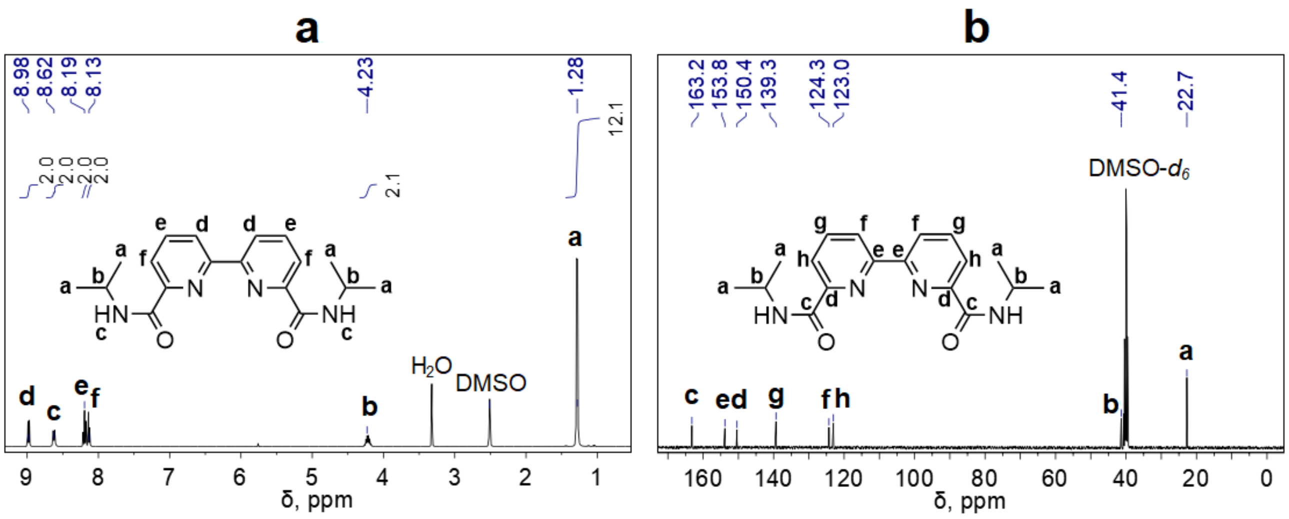

Figure S1. ^{1}H (a) and ^{13}C NMR (b) spectrum of BDCA ligand.

2. X-ray diffraction

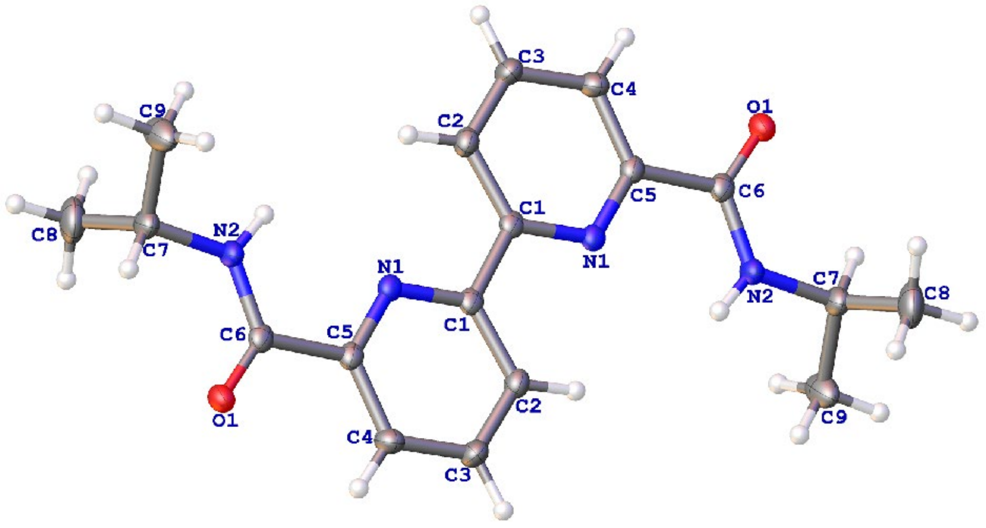

Figure S2. Molecular structures BDCA with thermal ellipsoids shown at the 50% probability level. The molecules of solvents are omitted for better representability.

Table S1. Crystallographic information for structures of BDCA, $[Eu(BDCA)_2(H_2O)]Cl_3$, and $[Tb(BDCA)_2(H_2O)]Cl_3$.

Identification code	BDCA	$[Eu(BDCA)_2(H_2O)]Cl_3$	$[Tb(BDCA)_2(H_2O)]Cl_3$
Empirical formula	$C_{18}H_{22}N_4O_2$	$C_{36}H_{58}Cl_3EuN_8O_{11}$	$C_{36}H_{58}Cl_3N_8O_{11}Tb$
Formula weight	326.39	1037.21	1044.17
Crystal system	monoclinic	monoclinic	monoclinic
Space group	$P2_1/c$	$P2/c$	$P2/c$
a, Å	9.2043(6)	11.2713(2)	11.2666(3)
b, Å	11.4313(5)	15.3222(2)	15.2986(3)
c, Å	9.6843(6)	14.2917(2)	14.2345(3)
α, °	90	90	90
β, °	116.612(8)	106.511(2)	106.335(3)
γ, °	90	90	90
Volume, Å^3	911.01(11)	2366.42(7)	2354.46(10)
Z	2	2	2
ρ_{calc}, g·cm^{-3}	1.190	1.456	1.473
μ, mm^{-1}	0.644	11.555	9.465
$F(000)$	348.0	1064.0	1068.0
Crystal size, mm^3	0.15×0.1×0.1	0.12×0.06×0.04	0.1×0.08×0.06
2Θ range for data collection, °	10.75 to 139.938	5.768 to 139.982	5.776 to 149.96
Index ranges	$-11 \le h \le 10$, $-13 \le k \le 13$, $-8 \le l \le 11$	$-13 \le h \le 13$, $-16 \le k \le 18$, $-17 \le l \le 17$	$-14 \le h \le 13$, $-19 \le k \le 15$, $-17 \le l \le 17$
Reflections collected	4303	17784	18261
Independent reflections	1723 [R_{int} = 0.1006, R_{sigma} = 0.0721]	4494 [R_{int} = 0.0746, R_{sigma} = 0.0496]	4819 [R_{int} = 0.0479, R_{sigma} = 0.0361]
Data/restraints/parameters	1723/0/111	4494/5/288	4819/7/284
Goodness-of-fit on F^2	1.058	1.034	1.034
Final R indexes [$I \ge 2\sigma(I)$]	R_1 = 0.0741, wR_2 = 0.1998	R_1 = 0.0312, wR_2 = 0.0812	R_1 = 0.0351, wR_2 = 0.0895
Final R indexes [all data]	R_1 = 0.0826, wR_2 = 0.2166	R_1 = 0.0317, wR_2 = 0.0816	R_1 = 0.0357, wR_2 = 0.0899
Largest diff. peak/hole, e·Å^{-3}	0.39/–0.43	0.69/–0.91	1.89/–1.11

3. Mass-spectra

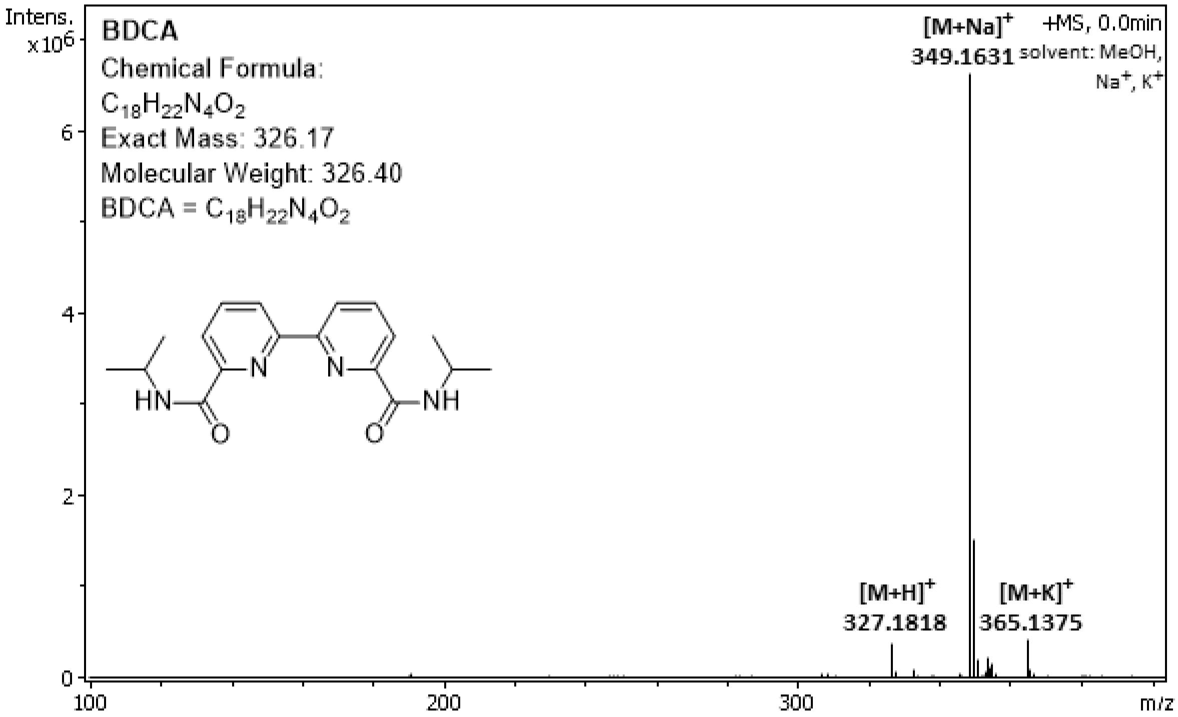

Figure S3. HRESIMS spectra of BDCA ligand.

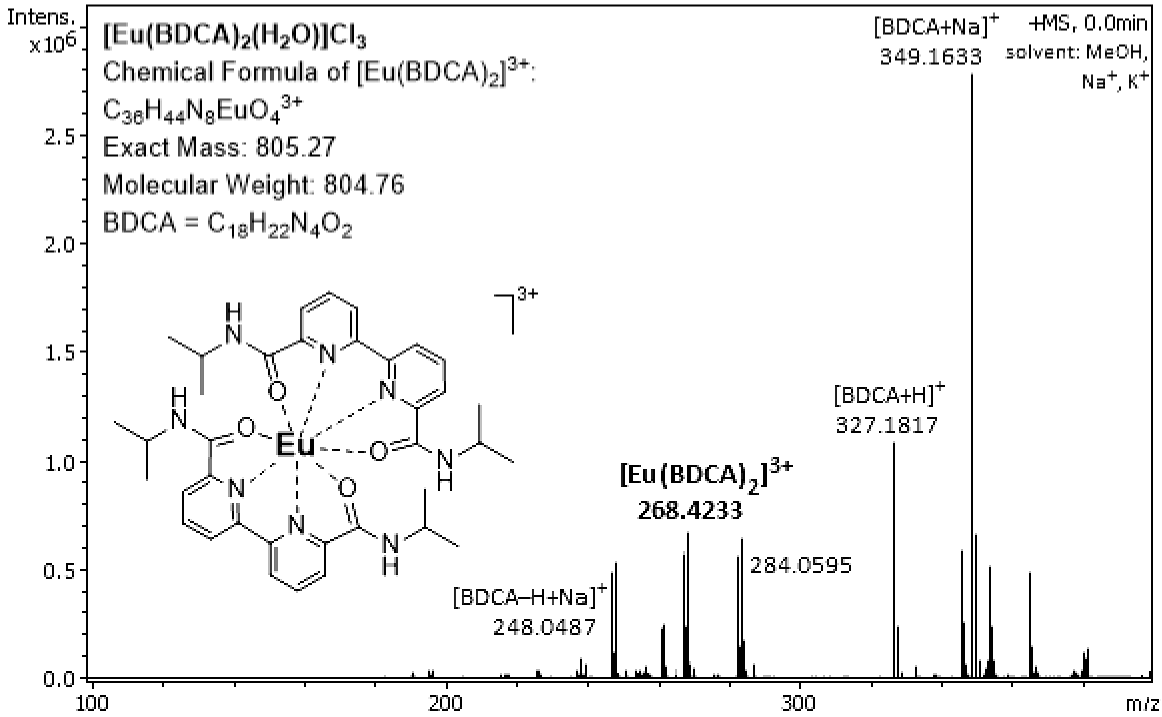

Figure S4. HRESIMS spectrum of [Eu(BDCA)$_2$(H$_2$O)]Cl$_3$ complex.

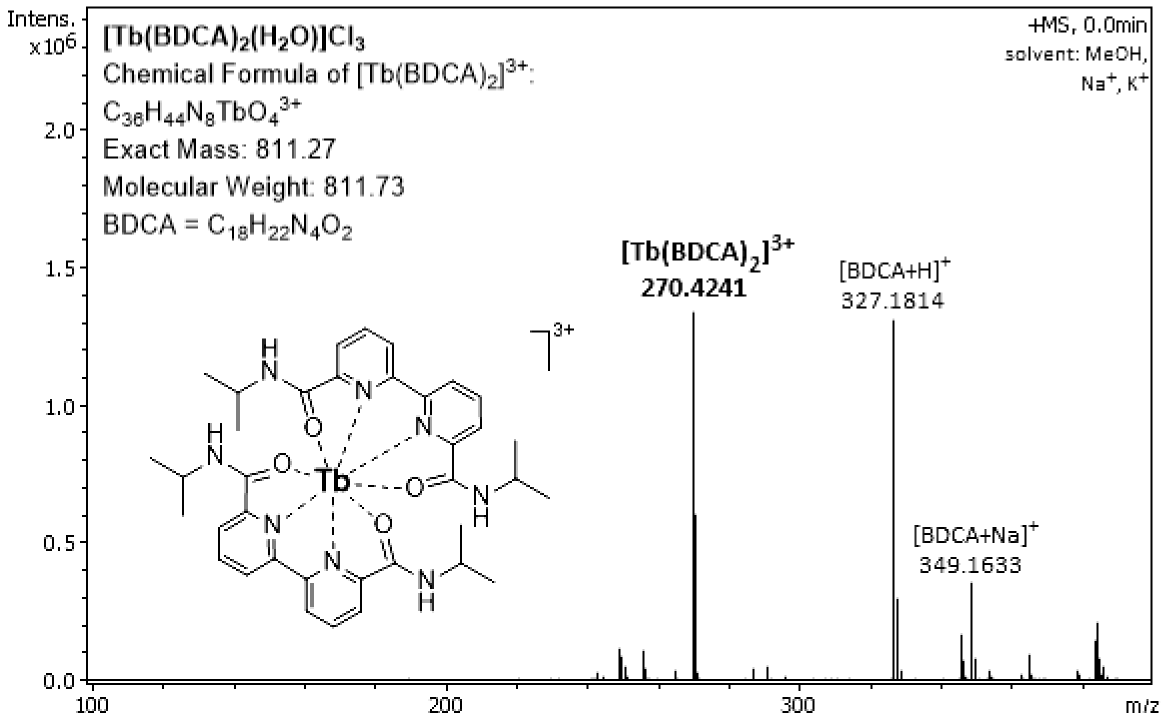

Figure S5. HRESIMS spectrum of $[Tb(BDCA)_2(H_2O)]Cl_3$ complex.

4. Theoretical calculations

4.1. Theoretical calculations of the coordination bonding energies

The QTAIM analysis demonstrates the presence of appropriate bond critical points (BCPs) (3, –1) for coordination bonds Ln–N$_{Bipy}$ and Ln–O in model structures [Eu(BDCA)$_2$(H_2O)]Cl_3 and [Tb(BDCA)$_2$(H_2O)]Cl_3 (Table S1). The low magnitude of the electron density (0.039–0.048 a.u.), positive values of the Laplacian of electron density (0.161–0.250 a.u.), and close to zero positive energy density (0.002–0.008 a.u.) in these BCPs are typical for closed shell contacts. The strength of coordination bonds Ln–N$_{Bipy}$ and Ln–O in [Eu(BDCA)$_2$(H_2O)]Cl_3 and [Tb(BDCA)$_2$(H_2O)]Cl_3 increases in the row: Ln–N$_{Bipy}$ (11.3–11.9 kcal·mol^{-1}) < Ln–H_2O (12.6–12.9 kcal·mol^{-1}) < Ln–O (15.1–16.0 kcal·mol^{-1}). The balance between the Lagrangian kinetic energy $G(\mathbf{r})$ and potential energy density $V(\mathbf{r})$ at the BCPs (3, –1) reveals the nature of these interactions, if the ratio $-G(\mathbf{r})/V(\mathbf{r}) > 1$ is satisfied, than the nature of appropriate interaction is purely non-covalent, in case the $-G(\mathbf{r})/V(\mathbf{r}) < 1$ some covalent component takes place [1]; based on this criterion one can state that all contacts listed in Table S1 are non-covalent. The Laplacian of electron density is typically decomposed into the sum of contributions along the three principal axes of maximal variation, giving the three eigenvalues of the Hessian matrix (λ_1, λ_2 and λ_3), and the sign of λ_2 can be utilized to distinguish bonding (attractive, $\lambda_2 < 0$) interactions from non-bonding ones (repulsive, $\lambda_2 > 0$) [2,3]. Thus, all coordination bonds Ln–N$_{Bipy}$ and Ln–O in model structures [Eu(BDCA)$_2$(H_2O)]Cl_3 and [Tb(BDCA)$_2$(H_2O)]Cl_3 are attractive (Table S1).

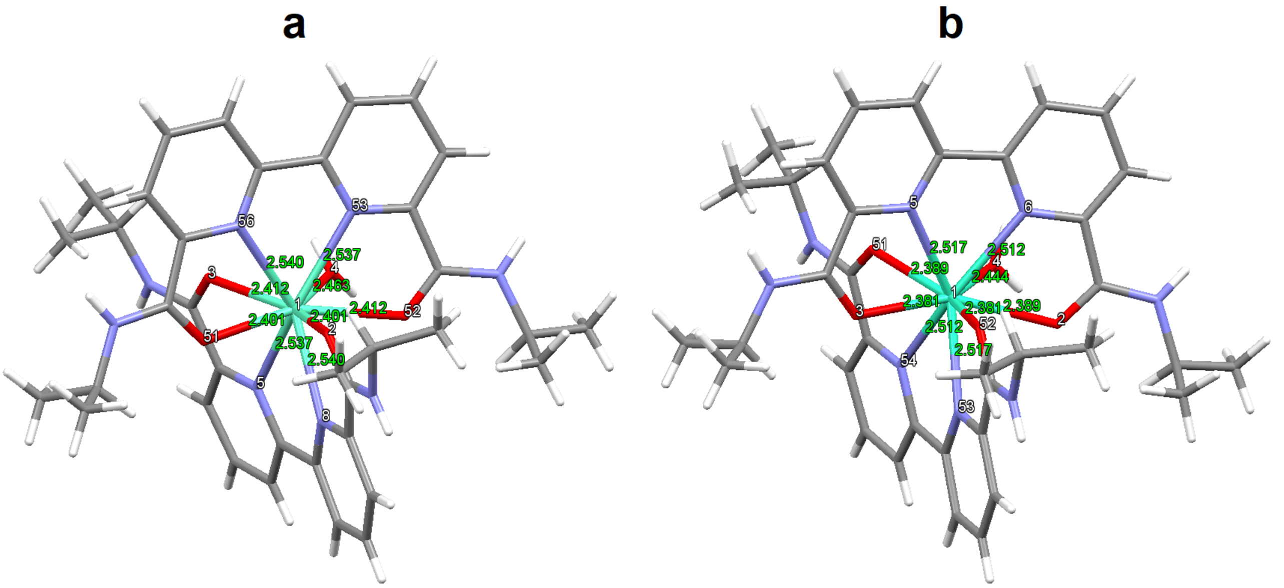

Figure S6. Model structures [Eu(BDCA)$_2$(H_2O)]Cl_3 (**a**) and [Tb(BDCA)$_2$(H_2O)]Cl_3 (**b**). Length of coordination bonds Ln–O and Ln–N$_{Bipy}$ (Ln = Eu, Tb) are indicated in Å.

Table S2. Values of the density of all electrons — *ρ(r)*, Laplacian of electron density – *∇²ρ(r)* and appropriate λ_2 eigenvalues, energy density — H_b, potential energy density — *V(r)*, Lagrangian kinetic energy — *G(r)*, and electron localization function — *ELF* (a.u.) at the bond critical points (3, –1), corresponding to coordination bonds Ln–O and Ln–N_{Bipy} (Ln = Eu, Tb) in model structures [Eu(BDCA)$_2$(H$_2$O)]Cl$_3$ and [Tb(BDCA)$_2$(H$_2$O)]Cl$_3$, as well as estimated energies for these contacts E_{int} (kcal·mol^{-1}) and bond lengths — *l* (Å).

Contact*	*ρ(r)*	*∇²ρ(r)*	λ_2	H_b	*V(r)*	*G(r)*	*ELF*	E_{int}	*l*, Å
				**[Eu(BDCA)$_2$(H$_2$O)]Cl$_3$**					
Eu1–O4	0.039	0.209	–0.039	0.006	–0.040	0.046	0.071	12.6	2.463
Eu1–O51	0.048	0.229	–0.048	0.003	–0.050	0.053	0.105	15.7	2.401
Eu1–O2	0.048	0.229	–0.048	0.003	–0.050	0.053	0.105	15.7	2.401
Eu1–O3	0.047	0.225	–0.047	0.005	–0.048	0.052	0.101	15.1	2.412
Eu1–O52	0.047	0.225	–0.047	0.005	–0.048	0.052	0.101	15.1	2.412
Eu1–N56	0.042	0.161	–0.042	0.002	–0.036	0.038	0.125	11.3	2.540
Eu1–N8	0.042	0.161	–0.042	0.002	–0.036	0.038	0.125	11.3	2.540
Eu1–N53	0.042	0.165	–0.042	0.002	–0.037	0.039	0.123	11.6	2.537
Eu1–N5	0.042	0.165	–0.042	0.002	–0.037	0.039	0.123	11.6	2.537
				**[Tb(BDCA)$_2$(H$_2$O)]Cl$_3$**					
Tb1–O4	0.039	0.225	–0.039	0.008	–0.041	0.049	0.065	12.9	2.444
Tb1–O3	0.048	0.250	–0.048	0.005	–0.051	0.056	0.094	16.0	2.381
Tb1–O52	0.048	0.250	–0.048	0.005	–0.051	0.056	0.094	16.0	2.381
Tb1–O51	0.047	0.243	–0.047	0.006	–0.048	0.054	0.090	15.1	2.389
Tb1–O2	0.047	0.243	–0.047	0.006	–0.048	0.054	0.090	15.1	2.389
Tb1–N5	0.041	0.174	–0.041	0.003	–0.037	0.040	0.111	11.6	2.517
Tb1–N53	0.041	0.174	–0.041	0.003	–0.037	0.040	0.111	11.6	2.517
Tb1–N6	0.042	0.179	–0.042	0.003	–0.038	0.041	0.109	11.9	2.512
Tb1–N54	0.042	0.179	–0.042	0.003	–0.038	0.041	0.109	11.9	2.512

* Numeration of atoms corresponds to their ordering in model structures [Eu(BDCA)$_2$(H$_2$O)]Cl$_3$ and [Tb(BDCA)$_2$(H$_2$O)]Cl$_3$ (see Figure S6),
$E_{int} \approx -V(\mathbf{r})/2$ [4].

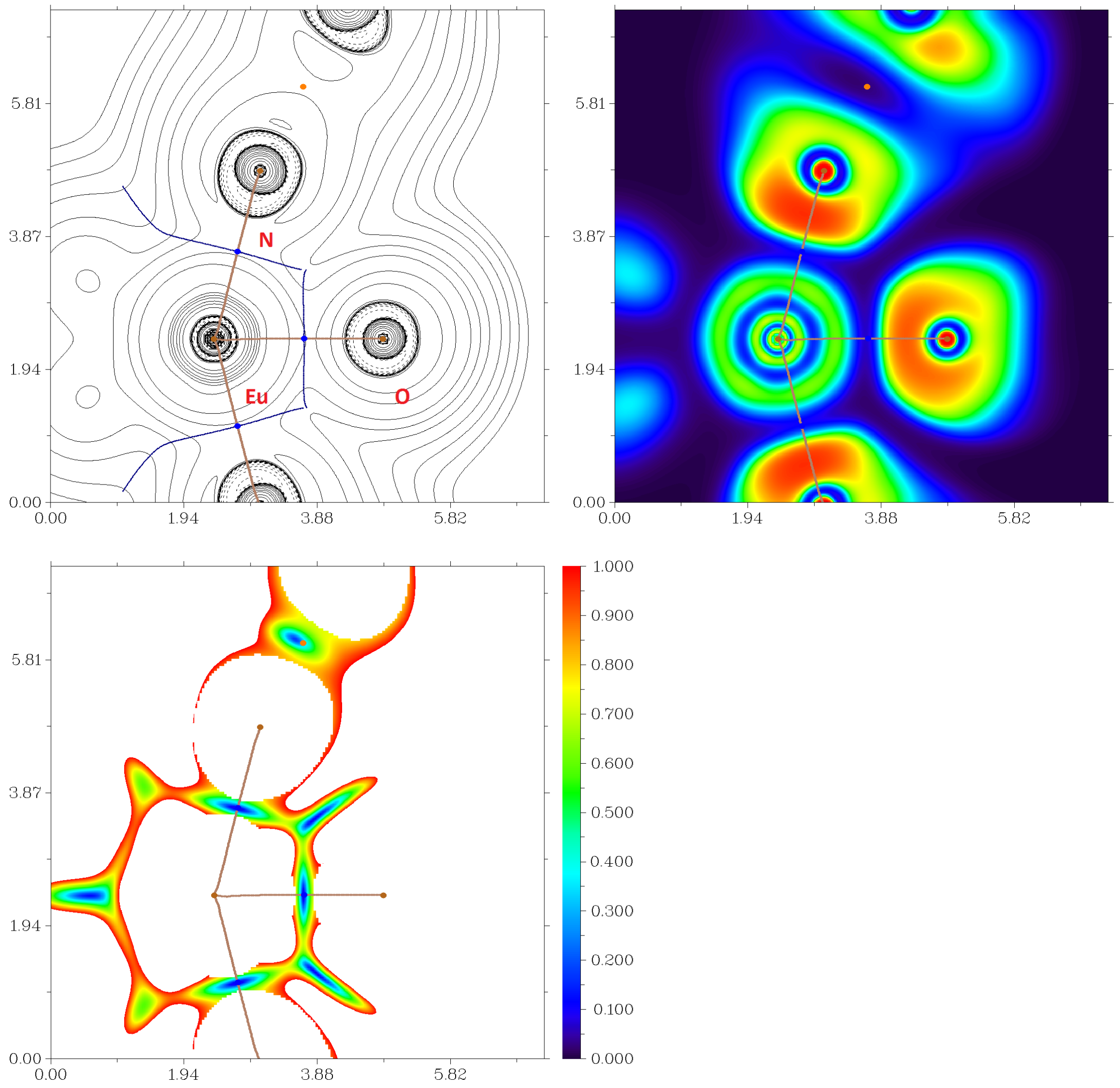

Figure S7. Contour line diagram of the Laplacian of electron density distribution $\nabla^2\varrho(r)$, bond paths, and selected zero-flux surfaces (left panel), visualization of electron localization function (ELF, center panel) and reduced density gradient (RDG, right panel) analyses referring to coordination bonds Eu–O and Eu–N in model structure $[Eu(BDCA)_2(H_2O)]Cl_3$. Bond critical points (3, –1) are shown in blue, nuclear critical points (3, –3) — in pale brown, ring critical points (3, +1) — in orange, bond paths are shown as pale brown lines, length units – Å, and the color scale for the ELF and RDG maps is presented in a.u.

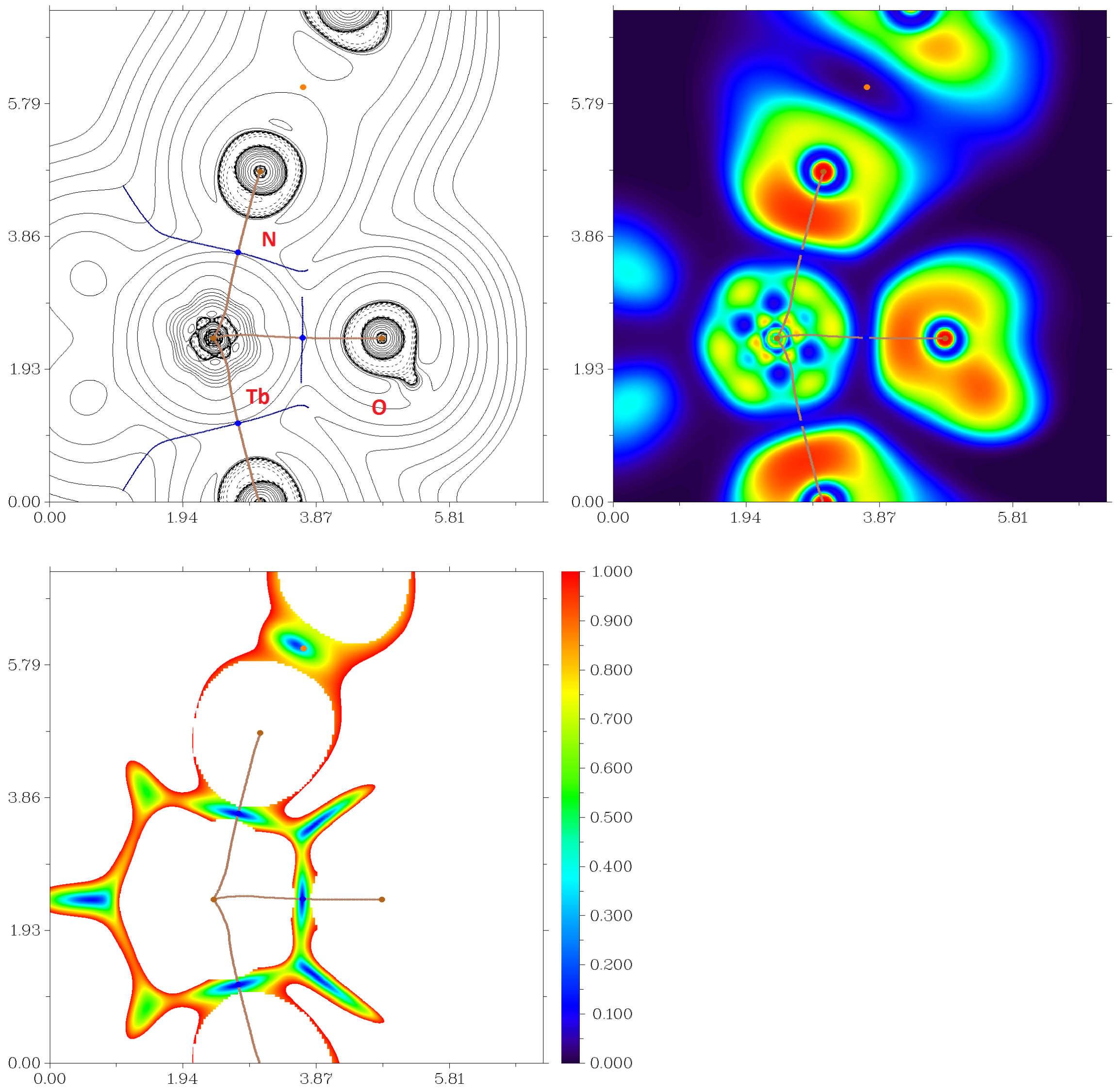

Figure S8. Contour line diagram of the Laplacian of electron density distribution $\nabla^2\rho(r)$, bond paths, and selected zero-flux surfaces (left panel), visualization of electron localization function (ELF, center panel) and reduced density gradient (RDG, right panel) analyses referring to coordination bonds Tb–O and Tb–N in model structure $[Tb(BDCA)_2(H_2O)]Cl_3$. Bond critical points (3, –1) are shown in blue, nuclear critical points (3, –3) – in pale brown, ring critical points (3, +1) – in orange, bond paths are shown as pale brown lines, length units – Å, and the color scale for the ELF and RDG maps is presented in a.u.

4.2. Theoretical calculations of HOMO–LUMO energy gaps

The HOMO–LUMO energy gap is the difference between the energies of HOMO and LUMO. For [Eu(BDCA)$_2$(H_2O)]Cl_3, E_{HOMO} = –16.43 eV, E_{LUMO} = –8.93 eV; for [Tb(BDCA)$_2$(H_2O)]Cl_3, E_{HOMO} = –16.44 eV, E_{LUMO} = –8.27 eV, respectively.

4.3. Theoretical modeling of UV-vis absorption spectra

Results of TD-DFT calculations at the CAM-B3LYP/CEP-121G level of theory for model structure [Eu(BDCA)$_2$(H_2O)]Cl_3 (Figure S9) reveal that experimentally observed picks at 300 and 290 nm in the recorded UV-Vis spectra clearly associated with the ligand-to-metal charge transfer (LMCT).

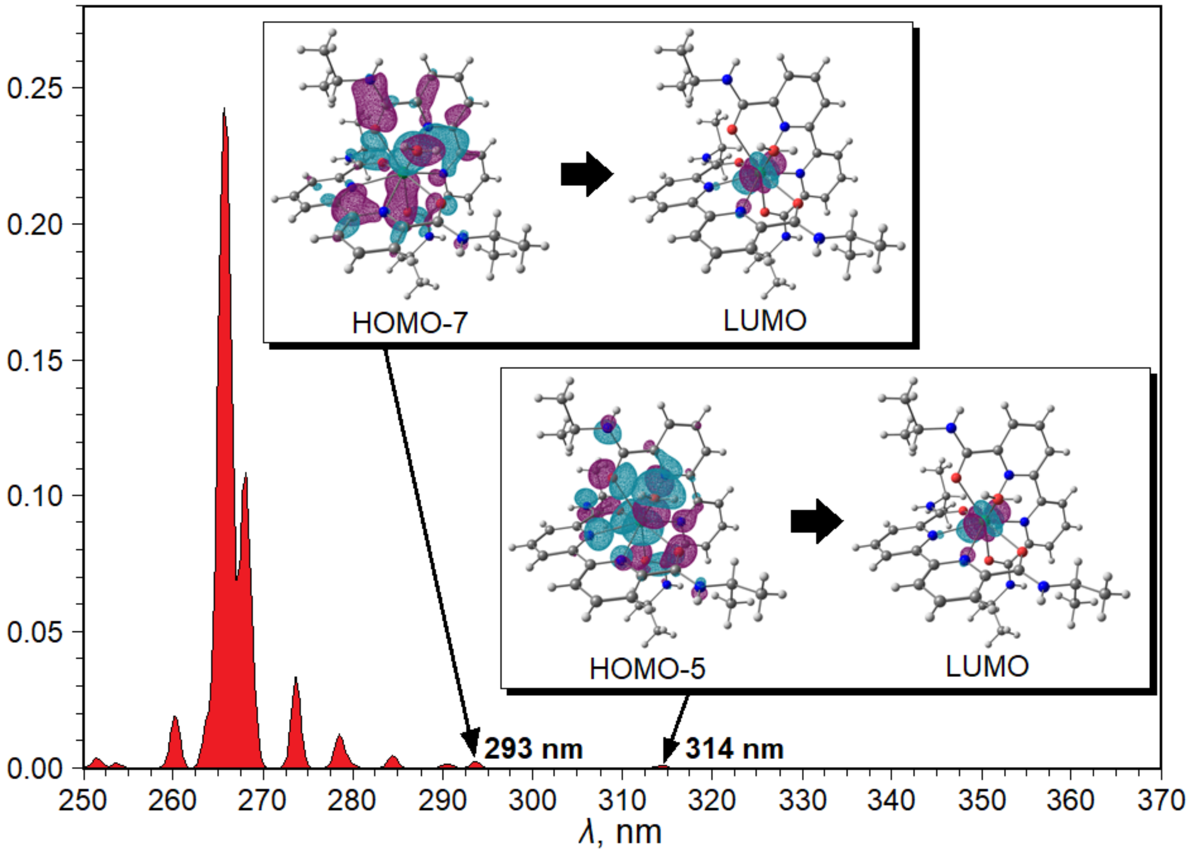

Figure S9. TD-DFT simulated UV-Vis spectrum of model structure [Eu(BDCA)$_2$(H_2O)]Cl_3.

4.4. Computational details

The DFT calculations were carried out using the dispersion-corrected hybrid functional ωB97XD [5] with the help of Gaussian-09 program package [6] based on the experimental X-ray structures of [Eu(BDCA)$_2$(H_2O)]Cl_3 and [Tb(BDCA)$_2$(H_2O)]Cl_3. The Douglas–Kroll–Hess 2nd order scalar relativistic calculations requested relativistic core Hamiltonian were carried out using the DZP-DKH basis sets [7–10] for all atoms. The topological analysis of the electron density distribution was performed by using the Multiwfn program (version 3.7) [11]. The Cartesian atomic coordinates for model structures are presented in the Table S2 and in the attached XYZ-files.

Table S3. Cartesian atomic coordinates for model structures [Eu(BDCA)$_2$(H$_2$O)]Cl$_3$ and [Tb(BDCA)$_2$(H$_2$O)]Cl$_3$.

Atom	*X*	*Y*	*Z*
		**[Eu(BDCA)$_2$(H$_2$O)]Cl$_3$**	
Eu	4.620227	3.655264	3.425596
O	5.764424	5.565023	2.527679
O	2.536633	2.842728	4.328994
O	4.620227	1.192067	3.425596
N	4.666009	2.985071	5.872020
N	1.340112	1.666136	5.833105
H	1.336462	1.217058	6.589367
N	6.474228	4.611369	4.874760
N	7.467598	7.043615	2.451357
H	8.198338	7.345432	2.838449
C	5.767562	3.144115	6.623733
C	3.656082	2.243170	6.335983
C	6.782224	6.059930	3.029597
C	7.277230	5.555830	4.355988
C	6.764959	4.100221	6.087969
C	2.452619	2.256960	5.426144
C	5.909625	2.508244	7.855577
H	6.699003	2.623467	8.372088
C	7.879165	4.520049	6.804604
H	8.070111	4.143874	7.655632
C	3.717265	1.576654	7.555495
H	2.988059	1.051731	7.864511
C	0.103933	1.752860	5.026035
H	0.352005	1.703951	4.058838
C	8.706771	5.486880	6.267471
H	9.475049	5.779319	6.743793
C	8.402361	6.029286	5.020554
H	8.948983	6.704780	4.635983
C	7.032584	7.635052	1.181146
H	6.152515	7.230791	0.931077

C	4.882089	1.706893	8.310496
H	4.968820	1.243611	9.135900
C	-0.560383	3.084359	5.282269
H	–0.761435	3.171098	6.238339
H	–1.393433	3.138967	4.768443
H	0.040275	3.808226	5.008537
C	–0.785423	0.583776	5.349411
H	–0.362177	–0.244159	5.040395
H	–1.649463	0.696287	4.900740
H	–0.924705	0.537304	6.318663
C	8.030771	7.304093	0.102768
H	8.911214	7.651830	0.354234
H	7.746792	7.712920	–0.741874
H	8.082368	6.331409	–0.007112
C	6.835732	9.132031	1.366128
H	6.143785	9.289697	2.042477
H	6.558829	9.533166	0.516210
H	7.677779	9.538422	1.660318
H	5.193319	0.679172	3.831762
H	3.930695	0.680750	3.284900
O	3.476030	5.565023	4.323513
O	6.703821	2.842728	2.522198
N	4.574445	2.985071	0.979172
N	7.900341	1.666136	1.018087
H	7.903991	1.217058	0.261825
N	2.766226	4.611369	1.976432
N	1.772855	7.043615	4.399836
H	1.042115	7.345432	4.012743
C	3.472891	3.144115	0.227460
C	5.584372	2.243170	0.515210
C	2.458230	6.059930	3.821595
C	1.963223	5.555830	2.495204
C	2.475494	4.100221	0.763223
C	6.787835	2.256960	1.425048
C	3.330829	2.508244	–1.004385

H	2.541451	2.623467	−1.520896
C	1.361289	4.520049	0.046588
H	1.170342	4.143874	−0.804440
C	5.523189	1.576654	−0.704303
H	6.252394	1.051731	−1.013319
C	9.136521	1.752860	1.825158
H	8.888449	1.703951	2.792354
C	0.533682	5.486880	0.583722
H	−0.234595	5.779319	0.107399
C	0.838092	6.029286	1.830639
H	0.291470	6.704780	2.215210
C	2.207870	7.635052	5.670047
H	3.087939	7.230791	5.920115
C	4.358364	1.706893	−1.459304
H	4.271634	1.243611	−2.284708
C	9.800836	3.084359	1.568923
H	10.001889	3.171098	0.612853
H	10.633886	3.138967	2.082749
H	9.200179	3.808226	1.842656
C	10.025876	0.583776	1.501781
H	9.602630	−0.244159	1.810798
H	10.889917	0.696287	1.950452
H	10.165158	0.537304	0.532529
C	1.209683	7.304093	6.748424
H	0.329239	7.651830	6.496958
H	1.493661	7.712920	7.593067
H	1.158086	6.331409	6.858304
C	2.404721	9.132031	5.485065
H	3.096669	9.289697	4.808715
H	2.681625	9.533166	6.334982
H	1.562675	9.538422	5.190874
**$[Tb(BDCA)_2(H_2O)]Cl_3$**			
Tb	2.630678	11.303218	10.244929
O	4.698846	10.493004	9.365914

O	1.506795	13.189382	11.165743
O	2.630678	8.858960	10.244929
N	0.779476	12.243470	8.822933
N	2.574295	10.623501	7.827126
N	5.899817	9.313482	7.870701
H	5.903494	8.859970	7.116619
N	–0.186748	14.676712	11.239370
H	–0.917456	14.975448	10.850732
C	3.585903	9.884578	7.358591
C	1.475168	10.782759	7.075831
C	3.529205	9.223526	6.137395
H	4.261432	8.704276	5.826865
C	1.335395	10.144502	5.838244
H	0.545864	10.256533	5.322746
C	–0.637752	12.157797	6.892788
H	–0.835135	11.781299	6.043320
C	4.784254	9.900795	8.271073
C	0.487344	13.697143	10.661556
C	0.481523	11.740452	7.609933
C	7.138918	9.405579	8.667210
H	6.895472	9.365987	9.636776
C	–0.013326	13.191065	9.340643
C	–1.454008	13.126199	7.440550
H	–2.223872	13.422716	6.970008
C	–1.145236	13.663180	8.678138
H	–1.691024	14.336073	9.067322
C	2.363680	9.348974	5.382003
H	2.278824	8.888196	4.556261
C	0.241731	15.283301	12.502911
H	1.123852	14.884880	12.755770
C	8.021988	8.224527	8.348934
H	7.591582	7.401141	8.659233
H	8.885977	8.331801	8.798823
H	8.162873	8.174424	7.379873
C	7.801797	10.727378	8.396744

H	8.086702	10.764630	7.460016
H	8.582695	10.827339	8.980254
H	7.167137	11.453572	8.573926
C	–0.752617	14.952852	13.592972
H	–1.634792	15.301935	13.345755
H	–0.463078	15.363665	14.434040
H	–0.805035	13.981238	13.703972
C	0.434832	16.771855	12.313039
H	1.122320	16.928819	11.632775
H	0.715446	17.176059	13.160649
H	–0.409882	17.176442	12.022807
H	3.321125	8.386402	10.483745
H	2.249298	8.387289	9.621313
O	0.562511	10.493004	11.123944
O	3.754562	13.189382	9.324115
N	4.481881	12.243470	11.666925
N	2.687062	10.623501	12.662732
N	–0.638461	9.313482	12.619157
H	–0.642137	8.859970	13.373239
N	5.448105	14.676712	9.250488
H	6.178813	14.975448	9.639126
C	1.675454	9.884578	13.131267
C	3.786189	10.782759	13.414027
C	1.732152	9.223526	14.352463
H	0.999925	8.704276	14.662993
C	3.925962	10.144502	14.651615
H	4.715493	10.256533	15.167112
C	5.899109	12.157797	13.597070
H	6.096492	11.781299	14.446538
C	0.477103	9.900795	12.218785
C	4.774013	13.697143	9.828302
C	4.779834	11.740452	12.879925
C	–1.877561	9.405579	11.822648
H	–1.634115	9.365987	10.853082
C	5.274683	13.191065	11.149215

C	6.715365	13.126199	13.049308
H	7.485229	13.422716	13.519850
C	6.406592	13.663180	11.811720
H	6.952381	14.336073	11.422536
C	2.897677	9.348974	15.107855
H	2.982533	8.888196	15.933597
C	5.019626	15.283301	7.986947
H	4.137505	14.884880	7.734088
C	–2.760631	8.224527	12.140924
H	–2.330225	7.401141	11.830625
H	–3.624620	8.331801	11.691035
H	–2.901516	8.174424	13.109985
C	–2.540440	10.727378	12.093114
H	–2.825345	10.764630	13.029842
H	–3.321338	10.827339	11.509604
H	–1.905780	11.453572	11.915932
C	6.013974	14.952852	6.896886
H	6.896149	15.301935	7.144103
H	5.724435	15.363665	6.055819
H	6.066392	13.981238	6.785886
C	4.826525	16.771855	8.176819
H	4.139037	16.928819	8.857083
H	4.545911	17.176059	7.329209
H	5.671239	17.176442	8.467051

5. Luminescent properties

Excitation spectra of $[Eu(BDCA)_2(H_2O)]Cl_3$ and $[Tb(BDCA)_2(H_2O)]Cl_3$ were measured at emission wavelengths of 617 nm and 554 nm, respectively. Both $[Eu(BDCA)_2(H_2O)]Cl_3$ and $[Tb(BDCA)_2(H_2O)]Cl_3$ show a broad excitation peak centered at 335 nm (Figure S10, a, b). However, for $[Eu(BDCA)_2(H_2O)]Cl_3$ we observe a signal with low intensity at 400 nm, which could correspond to the ligand energy transition $S_1 \rightarrow T_1$ (Figure S10, a). After encapsulation in PEG excitation spectra of $[Eu(BDCA)_2(H_2O)]Cl_3$ and $[Tb(BDCA)_2(H_2O)]Cl_3$ show a narrow peak at 320 nm (Figure S10, c, d). This could be explained by influence of the polymer matrix (PEG) [12].

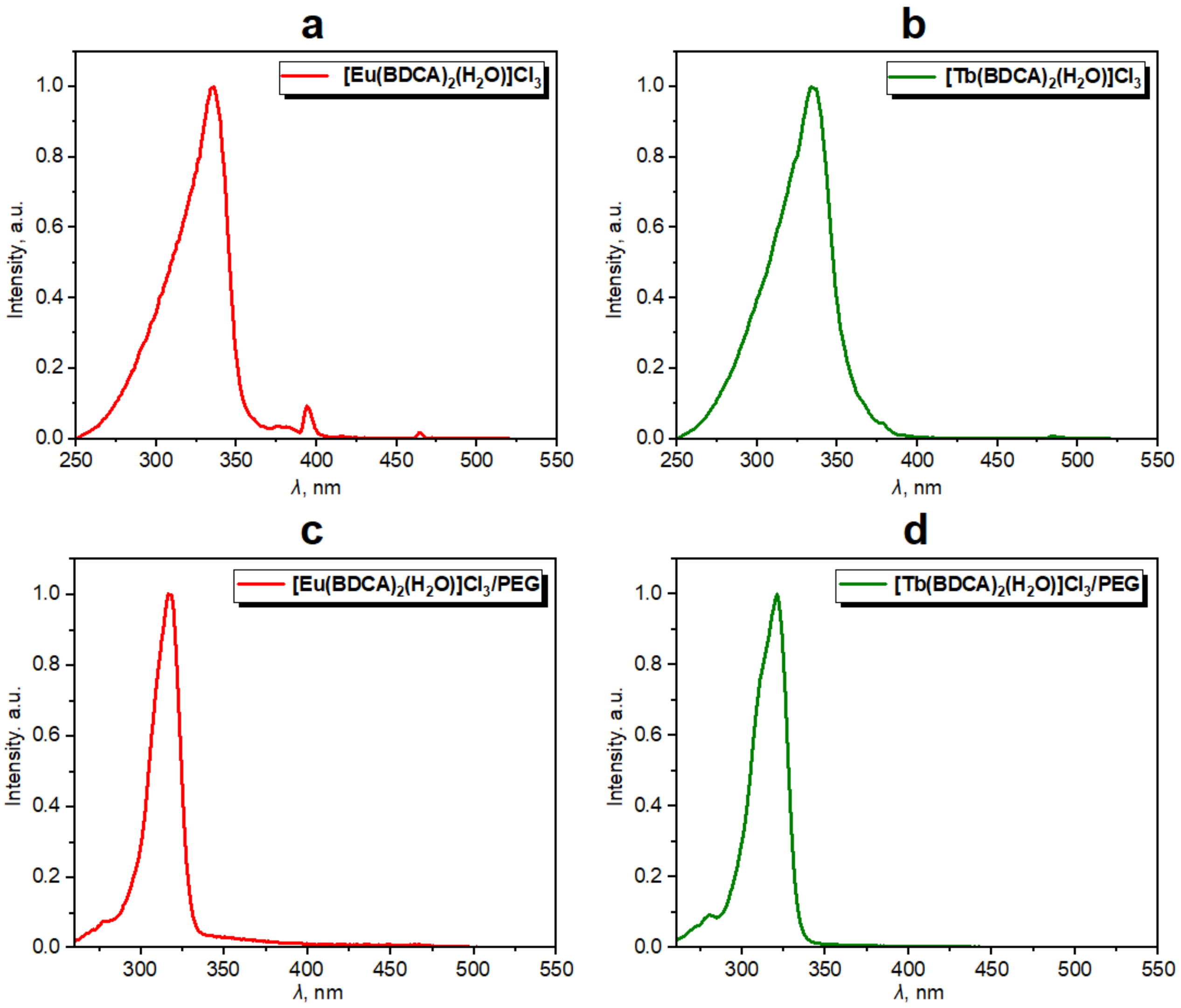

Figure S10. Excitation spectra of raw $[Eu(BDCA)_2(H_2O)]Cl_3$ (**a**), $[Tb(BDCA)_2(H_2O)]Cl_3$ (**b**), and the encapsulated complexes in PEG (**c**, **d**).

PL lifetime (Figure S11) for both raw and encapsulated in PEG $[Eu(BDCA)_2(H_2O)]Cl_3$ and $[Tb(BDCA)_2(H_2O)]Cl_3$ were measured at emission wavelengths 617 nm and 554 nm, respectively. Excitation wavelength was 335 nm for the raw and 320 nm for the encapsulated complexes.

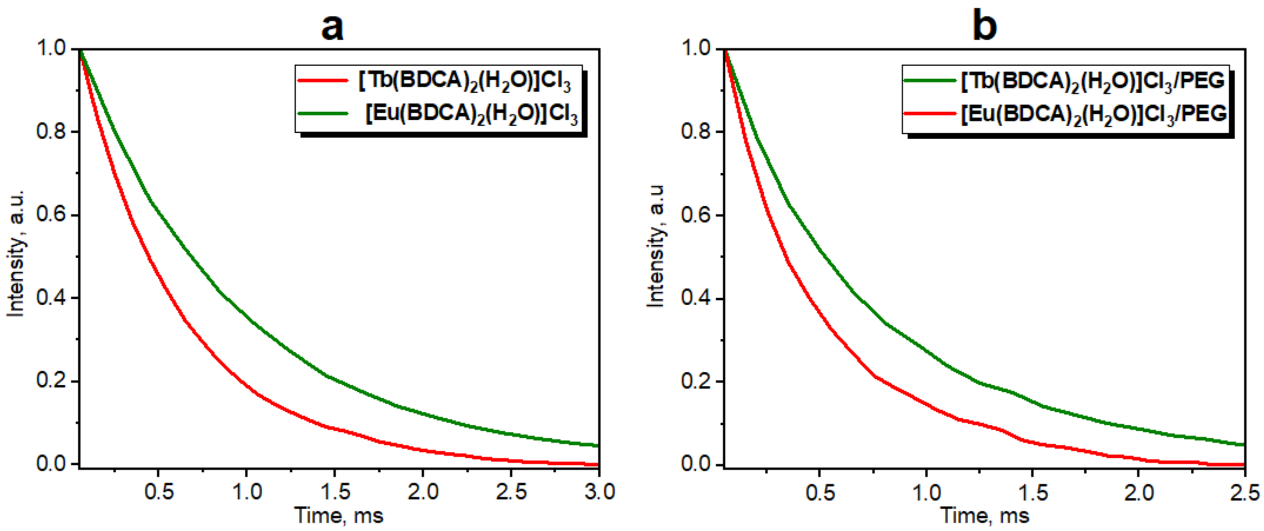

Figure S11. Luminescence lifetime decay for $[Eu(BDCA)_2(H_2O)]Cl_3$ (**a**), $[Tb(BDCA)_2(H_2O)]Cl_3$ (**b**), and the encapsulated complexes in PEG (**c**, **d**).

Lifetime value was estimated by exponential approximation according to the Equation (1):

$$y = A_1 \cdot exp(-x/t_1) + y_0, \tag{1}$$

where $y_0 = 0$, t_1 — decay time constant, A_1 — corresponding intensity.

6. References

1. Espinosa, E.; Alkorta, I.; Elguero, J.; Molins, E. From Weak to Strong Interactions: A Comprehensive Analysis of the Topological and Energetic Properties of the Electron Density Distribution Involving X–H···F–Y Systems. *The Journal of Chemical Physics* **2002**, *117*, 5529–5542, doi:10.1063/1.1501133.
2. Johnson, E.R.; Keinan, S.; Mori-Sánchez, P.; Contreras-García, J.; Cohen, A.J.; Yang, W. Revealing Noncovalent Interactions. *J. Am. Chem. Soc.* **2010**, *132*, 6498–6506, doi:10.1021/ja100936w.
3. Contreras-García, J.; Johnson, E.R.; Keinan, S.; Chaudret, R.; Piquemal, J.-P.; Beratan, D.N.; Yang, W. NCIPLOT: A Program for Plotting Noncovalent Interaction Regions. *J. Chem. Theory Comput.* **2011**, *7*, 625–632, doi:10.1021/ct100641a.
4. Espinosa, E.; Molins, E.; Lecomte, C. Hydrogen Bond Strengths Revealed by Topological Analyses of Experimentally Observed Electron Densities. *Chemical Physics Letters* **1998**, *285*, 170–173, doi:10.1016/S0009-2614(98)00036-0.
5. Chai, J.-D.; Head-Gordon, M. Long-Range Corrected Hybrid Density Functionals with Damped Atom–Atom Dispersion Corrections. *Phys. Chem. Chem. Phys.* **2008**, *10*, 6615, doi:10.1039/b810189b.
6. Frisch, M.J.; Trucks, G.W.; Schlegel, H.B.; Scuseria, G.E.; Robb, M.A.; Cheeseman, J.R.; Scalmani, G.; Barone, V.; Mennucci, B.; Petersson, G.A.; et al. *Gaussian 09*; Revision C.01.; Gaussian Inc.: Wallingford, CT, 2010;
7. Barros, C.L.; de Oliveira, P.J.P.; Jorge, F.E.; Canal Neto, A.; Campos, M. Gaussian Basis Set of Double Zeta Quality for Atoms Rb through Xe: Application in Non-Relativistic and Relativistic Calculations of Atomic and Molecular Properties. *Molecular Physics* **2010**, *108*, 1965–1972, doi:10.1080/00268976.2010.499377.
8. Jorge, F.E.; Canal Neto, A.; Camiletti, G.G.; Machado, S.F. Contracted Gaussian Basis Sets for Douglas–Kroll–Hess Calculations: Estimating Scalar Relativistic Effects of Some Atomic and Molecular Properties. *The Journal of Chemical Physics* **2009**, *130*, 064108, doi:10.1063/1.3072360.
9. Canal Neto, A.; Jorge, F.E. All-Electron Double Zeta Basis Sets for the Most Fifth-Row Atoms: Application in DFT Spectroscopic Constant Calculations. *Chemical Physics Letters* **2013**, *582*, 158–162, doi:10.1016/j.cplett.2013.07.045.
10. de Berrêdo, R.C.; Jorge, F.E. All-Electron Double Zeta Basis Sets for Platinum: Estimating Scalar Relativistic Effects on Platinum(II) Anticancer Drugs. *Journal of Molecular Structure: THEOCHEM* **2010**, *961*, 107–112, doi:10.1016/j.theochem.2010.09.007.
11. Lu, T.; Chen, F. Multiwfn: A Multifunctional Wavefunction Analyzer. *J. Comput. Chem.* **2012**, *33*, 580–592, doi:10.1002/jcc.22885.
12. *Luminescence of Lanthanide Ions in Coordination Compounds and Nanomaterials*; de Bettencourt-Dias, A., Ed.; John Wiley & Sons Ltd: Chichester, United Kingdom, 2014; ISBN 978-1-118-68276-0.
